# Locomotor Muscles in COPD: The Rationale for Rehabilitative Exercise Training

**DOI:** 10.3389/fphys.2019.01590

**Published:** 2020-01-14

**Authors:** Mathieu Marillier, Anne-Catherine Bernard, Samuel Vergès, J. Alberto Neder

**Affiliations:** ^1^Laboratory of Clinical Exercise Physiology, Kingston General Hospital, Queen’s University, Kingston, ON, Canada; ^2^HP2 Laboratory, INSERM, CHU Grenoble Alpes, Grenoble Alpes University, Grenoble, France

**Keywords:** chronic obstructive pulmonary disease, exercise training, muscle function, pulmonary rehabilitation, skeletal muscle

## Abstract

Exercise training as part of pulmonary rehabilitation is arguably the most effective intervention to improve tolerance to physical exertion in patients with chronic obstructive pulmonary disease (COPD). Owing to the fact that exercise training has modest effects on exertional ventilation, operating lung volumes and respiratory muscle performance, improving locomotor muscle structure and function are key targets for pulmonary rehabilitation in COPD. In the current concise review, we initially discuss whether patients’ muscles are exposed to deleterious factors. After presenting corroboratory evidence on this regard (e.g., oxidative stress, inflammation, hypoxemia, inactivity, and medications), we outline their effects on muscle macro- and micro-structure and related functional properties. We then finalize by addressing the potential beneficial consequences of different training strategies on these muscle-centered outcomes. This review provides, therefore, an up-to-date outline of the rationale for rehabilitative exercise training approaches focusing on the locomotor muscles in this patient population.

## Introduction

Exercise limitation in chronic obstructive pulmonary disease (COPD) is multi-factorial, including pulmonary gas exchange, mechanical and cardio-circulatory derangements (Neder et al., 2019). More recently, it has been recognized that impairment in peripheral muscle structure and function might also hold a relevant contributory role (Maltais et al., 2014). Importantly, locomotor muscle abnormalities, such as *quadriceps* weakness and atrophy, have been related to ominous clinical outcomes such as reduced quality of life and even mortality (Mostert et al., 2000; Swallow et al., 2007; Maltais et al., 2014).

Exercise training as part of pulmonary rehabilitation is the most effective strategy to improve tolerance to physical exertion and health-related quality of life in COPD (Troosters et al., 2005; Spruit et al., 2013, 2015). Despite a wide variability in the nature and composition of the rehabilitation programs, consistent improvements in exercise tolerance might be achieved (Lacasse et al., 2002; McCarthy et al., 2015). Consequently, participation in pulmonary rehabilitation is recommended as a pivotal intervention in symptomatic COPD regardless the severity of resting functional impairment (Singh et al., 2019). Beneficial changes in “respiratory responses” such as exertional ventilation (O’Donnell et al., 1998; Porszasz et al., 2005; Puente-Maestu et al., 2006), breathing pattern (O’Donnell et al., 1998), operating lung volumes (Porszasz et al., 2005; Puente-Maestu et al., 2006) and static respiratory muscle strength (Charususin et al., 2018; Langer et al., 2018) have been reported after training. These changes are, however, not particularly large and frequently inconsistent (Neder et al., 2019). For instance, improvement in dynamic inspiratory capacity at standardized exercise times averaged 0.1 to 0.4 L (Porszasz et al., 2005; Puente-Maestu et al., 2006) and were not always reported (O’Donnell et al., 1998). In this context, improving peripheral muscle structure and function represents a key target for pulmonary rehabilitation in COPD (Spruit et al., 2013).

The present concise review provides an up-to-date outline of the available literature supporting potential beneficial effects of pulmonary rehabilitation on locomotor muscle characteristics and function secondary to COPD. To achieve this goal, evidence is presented to answer three inter-related questions: *are patients’ skeletal muscles exposed to deleterious factors*? *is there evidence of locomotor muscle structural and/or functional abnormalities*? *can locomotor muscles abnormalities be reversed, in whole or in part, by rehabilitative exercise training*?

## Are Patients’ Skeletal Muscles Exposed to Deleterious Factors?

### Oxidative Stress

Oxidative stress reflects an imbalance between the rate at which reactive oxygen (O_2_) species (ROS, reactive chemical species containing O_2_) are produced and tissue antioxidant capacity. Oxidative stress can impair the structure and function of membrane lipids, proteins and deoxyribonucleic acid (DNA), potentially leading to cell injury. During exercise in patients with COPD, ROS are produced at a higher rate by muscle mitochondria which may lead to oxidative stress (Allaire et al., 2002; Couillard et al., 2003; Barreiro et al., 2009). Systemic and muscle oxidative stress, in turn, have been linked to poor muscle endurance in these patients (Couillard et al., 2002, 2003; Koechlin et al., 2004). For instance, Couillard et al. (2003) demonstrated an elevation in *quadriceps* lipid peroxidation and oxidized proteins after repeated knee extensions in COPD patients but not in controls. Other studies found that *quadriceps* muscle force was inversely related to the extent of local oxidative stress (Barreiro et al., 2009, 2010).

### Inflammation

Inflammation may lead to atrophy and impaired muscle regeneration (Maltais et al., 2014). Although this is not a consistent finding (Koechlin et al., 2005; Ryrso et al., 2018), expression of muscle tumor necrosis factor-α (TNF-α) was larger in COPD compared to controls (Montes de Oca et al., 2005). TNF-α could decrease muscle expression of insulin-like growth factor-I (IGF-I) and myogenic differentiation factor (MyoD) thereby inhibiting myogenic differentiation (Langen et al., 2004). However, a study reported similar content of interleukin (IL) 1β, IL-6, IL-8, and IL-18 as well as equal number of inflammatory cells in the *quadriceps femoris* of COPD patients compared to controls (Ryrso et al., 2018). Thus, the role of intra-muscular inflammation in the development of peripheral muscle dysfunction remains disputable in COPD.

### Hypoxia

Chronic hypoxemia but also tissue hypoxia have been associated with the extent of systemic inflammation (Takabatake et al., 2000; Pitsiou et al., 2002; Baldi et al., 2008) and may be an important factor contributing to loss of fat-free mass (Turan et al., 2011; Simoes and Vogiatzis, 2018). Chronic hypoxia has been associated with an overexpression of muscular DNA damage responses-1 (REDD1) in COPD (Favier et al., 2010), a negative regulator of mammalian target of rapamycin (mTOR) (Brugarolas et al., 2004). Therefore, chronic hypoxia downregulates muscle protein synthesis (Maltais et al., 2014). In addition, it has been shown to worsen exercise-induced muscle oxidative stress which may have negative consequences on *quadriceps* muscle endurance (see section “Oxidative Stress”) (Koechlin et al., 2005). Exercise-related hypoxemia may further aggravate exercise-induced oxidative stress and inflammatory response in COPD (Jammes et al., 2008; Slot et al., 2014). It also worsens skeletal muscle susceptibility to fatigue (Amann et al., 2010) through an impairment in muscle metabolism (Payen et al., 1993), O_2_ delivery and utilization (Maltais et al., 2001).

### Disuse

A systematic review including 47 studies found evidence that patients with COPD are physically less active in daily life compared to age and gender-matched controls (Bossenbroek et al., 2011). Muscle disuse secondary to years of physical inactivity [as a strategy to avoid facing exertional symptoms in particular (Guthrie et al., 2001; Lemmens et al., 2008; van Buul et al., 2017)] is, therefore, considered as a major contributor of muscle structural and functional abnormalities in COPD (Maltais et al., 2014; Jaitovich and Barreiro, 2018). It appears essential to emphasize that muscle disuse *per se* can trigger several alterations observed in the locomotor muscle of patients with COPD such as muscle atrophy or weakness (Booth and Gollnick, 1983). Nevertheless, some abnormalities [such as exercise-induced muscle oxidative stress, altered phenotypic expression of muscle myosin heavy chain or diverging pattern in muscle gene expression (Maltais et al., 1999; Couillard et al., 2003; Radom-Aizik et al., 2007)] are specifically observed in COPD but not in healthy subjects, even those who are extremely sedentary (Couillard and Prefaut, 2005; Maltais et al., 2014).

### Medications

Prolonged treatment with systemic corticosteroids worsens *quadriceps* muscle weakness in a dose-dependent fashion in COPD (Decramer et al., 1994). This might arise, at least partially, from negative morphological changes including preferential atrophy of type II fibers (Decramer et al., 1996). In fact, corticosteroids are known to inhibit protein synthesis (e.g., greater myostatin expression) and increase its degradation (e.g., low intra-muscle IGF-I levels) (Schakman et al., 2009).

### Summative Evidence

Collectively, oxidative stress and hypoxia (in more advanced disease) in the presence of muscle disuse in patients exposed to repeated courses of corticosteroids may indeed expose the skeletal muscles of COPD patients to a negative milieu. The role of inflammation remains elusive at this point in time.

## Is There Evidence of Locomotor Muscle Structural And/Or Functional Abnormalities?

### Structural Alterations

#### Muscle Mitochondria

**FIGURE 1:**
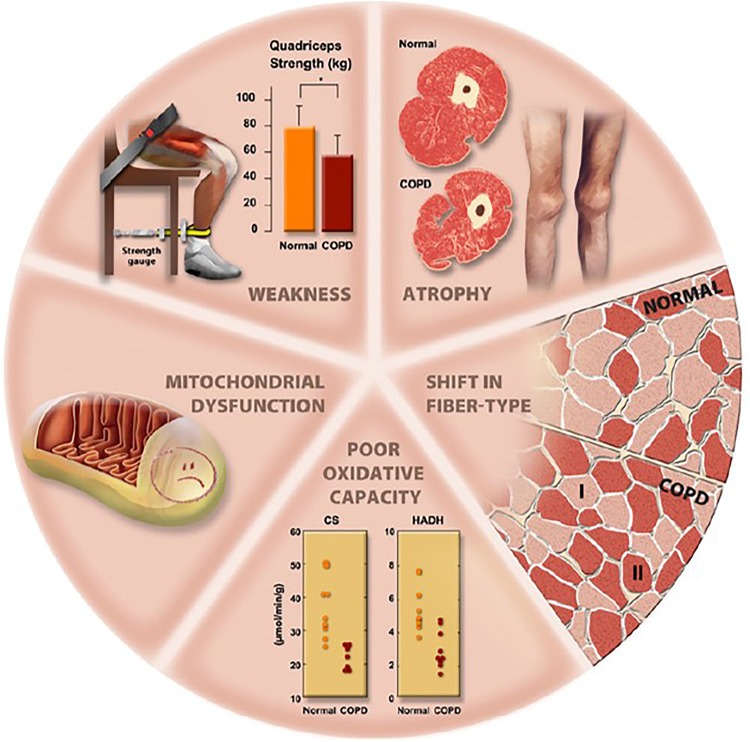
Overview of potential abnormalities in muscle structure and function in patients with COPD. Abbreviations: CS: citrate synthase; HADH: 3-hydroxyacyl CoA dehydrogenase. Reproduced, with permission from the publisher, from Maltais et al. (2014).

Several mitochondrial abnormalities have been described in the locomotor muscles of COPD patients [Figure 1, summarized in Taivassalo and Hussain (2016)]. It remains unclear, however, whether they reflect muscle disuse *per se* and/or a myopathic process (Maltais et al., 2014). Such alterations include lower mitochondrial density (Gosker et al., 2007) and lower oxidative enzyme activities (Figure 1), the latter leading to down-regulation of Krebs cycle and β-oxidation (Maltais et al., 2000; Puente-Maestu et al., 2009; Saey et al., 2011). Consequently, the efficiency of oxidative phosphorylation may be reduced (Picard et al., 2008; Naimi et al., 2011). Functionally, a lower oxidative enzyme activity (e.g., citrate synthase, CS) has been shown to correlate with impairments in muscle endurance (Allaire et al., 2004). Moreover, poorer mitochondrial synthesis has been consistently demonstrated in the locomotor muscles (Remels et al., 2007; Puente-Maestu et al., 2011; Konokhova et al., 2016). Higher mitochondrial degradation has also been reported (Guo et al., 2013; Leermakers et al., 2018) being related to muscle atrophy and lung function impairment (Guo et al., 2013). Konokhova et al. (2016) reported an elevated prevalence of mitochondrial DNA deletions which was in line with a higher proportion of oxidative-deficient fibers in the muscle of COPD patients compared to controls. Specifically, the presence of mitochondrial DNA deletions in COPD was related to a longer smoking history. In the same vein, Gifford et al. (2018) recently demonstrated a lower muscle CS activity and an altered mitochondrial respiration in COPD despite patients and controls had the same level of objective physical activity. Therefore, these results suggest that the low muscle oxidative capacity observed in COPD may be, at least in part, driven by a myopathic process specific to the disease. This may arise from COPD-related transcriptional perturbations evidenced in the *quadriceps* affecting muscle mitochondria (Willis-Owen et al., 2018). Overall, mitochondrial abnormalities may impair muscle oxidative capacity with a negative impact on endurance; furthermore, they may trigger protein breakdown thereby contributing to muscle atrophy and weakness (Figure 1; Gifford et al., 2015; Taivassalo and Hussain, 2016).

#### Muscle Protein Synthesis/Breakdown

Maintenance of muscle mass depends on the balance between protein synthesis and degradation. An important pathway for protein synthesis [Akt/mTOR pathway] is downregulated in the locomotor muscles of hypoxemic compared to normoxemic patients (see section “Hypoxia”) (Favier et al., 2010). A differential epigenetic profile (e.g., lower expression of IGF-I) has been also evidenced in patients with muscle weakness and atrophy (Puig-Vilanova et al., 2015). In fact, a biopsy-based study revealed a surge in markers for muscle protein degradation/synthesis and myogenesis in COPD compared to healthy subjects, suggesting greater muscle protein turnover in the former group (Constantin et al., 2013). Overall, there is a clear signal in favor of exaggerated muscle catabolism (Doucet et al., 2007; Plant et al., 2010; Constantin et al., 2013), despite lowering the influence of medication (systemic corticosteroids), aging and physical inactivity (Doucet et al., 2007), with the deterioration in cross-sectional area being particularly evident in type IIa and IIb fibers (Gosker et al., 2002).

#### Muscle Fiber Typing

Muscle fiber type distribution is shifted toward a more glycolytic profile: COPD patients typically exhibit a lower type I and greater type II fibers proportion compared to normal aging population (Figure 1; Maltais et al., 2014). Fiber type shift is particularly pronounced in COPD; for instance, while chronic sedentary subjects exhibit a one-third lower type I fiber proportion compared to active age-matched counterparts (Proctor et al., 1995; Houmard et al., 1998), a two-third smaller proportion is not unfrequently observed in patients (Couillard and Prefaut, 2005). Muscle fiber type shift appears heterogeneous across COPD as two phenotypes of patients showing different muscle histology (type I fiber proportion) have been identified (Gouzi et al., 2013a). Advanced muscle fiber type shift was characterized by an elevated muscle oxidative stress in particular. Type I fiber proportion, in turn, inversely correlates with the disease progression as indicated by the BODE index (body mass index, airflow obstruction, dyspnea and exercise capacity) (Vogiatzis et al., 2011). Recently, Kapchinsky et al. (2018) demonstrated that denervation of muscle fibers actually drives the fiber type shift observed in COPD. This was particularly evident in patients with low fat free mass, suggesting that denervation contributes to muscle atrophy in COPD. Evidence from a mouse model suggest a critical role of chronic tobacco smoke exposure in inducing denervation of muscle fibers (Kapchinsky et al., 2018).

#### Muscle Capillarization

Lesser capillary-to-fiber ratio has been found in the *quadriceps* (Jobin et al., 1998) and the *tibialis anterior* (Jatta et al., 2009) in COPD. This finding, however, is not universal: preserved capillarization has been also described across a wide range of COPD stages (Vogiatzis et al., 2011). Interestingly, COPD patients showing significant exercise-induced muscle fatigue had lower muscle capillarization compared to “non-fatiguers” (Saey et al., 2005), suggesting a mechanistic link between these phenomena.

### Muscle Function

#### Muscle Strength

Limb muscle weakness is a common finding in patients with COPD, particularly in the *quadriceps* (Figure 1; Maltais et al., 2014). *Quadriceps* weakness, in turn, has been negatively correlated with FEV_1_, suggesting a link with disease progression (Bernard et al., 1998; Seymour et al., 2010). A large retrospective study (Seymour et al., 2010), however, found substantial heterogeneity in the prevalence of *quadriceps* muscle weakness (defined specifically as observed values 1.645 standardized residuals below predicted values, previously determined in a group of 212 healthy participants): 28% in GOLD stages I-II and 38% in stage IV. This study and others (e.g., Clark et al., 2000) indicate that, despite appreciable variability, muscle weakness is not restricted to patients with severe airway obstruction. This is a critical observation since *quadriceps* muscle strength can predict mortality (Swallow et al., 2007) and is an important determinant of exercise tolerance (Gosselink et al., 1996) in COPD. Overall, *quadriceps* maximal voluntary contraction (MVC) is usually 25 to 30% lower in patients with COPD compared to controls (Bernard et al., 1998; Gosselink et al., 2000; Couillard et al., 2003; Debigare et al., 2003; Mador et al., 2003a; Allaire et al., 2004; Franssen et al., 2005; Man et al., 2005; Seymour et al., 2009, 2010). It is noteworthy that while some studies described a preserved *quadriceps* strength-thigh cross-sectional area or muscle mass ratio in patients with COPD (Bernard et al., 1998; Engelen et al., 2000; Couillard et al., 2003; Malaguti et al., 2011), others reported a larger impairment in muscle strength relative to mass (Debigare et al., 2003; Seymour et al., 2010). Comparing patients and controls of large dissimilar *quadriceps* muscle mass, Malaguti et al. (2006) found higher coefficients for allometric correction in the former group, i.e., more leg lean mass was required to generate a given functional output in patients. These results are consistent with the notion that factors other than simple atrophy (i.e., mass-independent mechanisms) play a role in explaining the COPD-related muscle weakness.

#### Muscle Endurance and Fatigability

Impaired *quadriceps* endurance is commonly seen in COPD; however, the extent of impairment varies substantially among studies [e.g., from ∼30% in Serres et al. (1998) to almost 80% in Coronell et al. (2004)]. Discrepancies between testing modalities [e.g., contraction regimen (isometric, isokinetic or isotonic), contraction type (repeated or sustained) or exercise intensity (% of MVC)] may contribute to these diverging results. While a large majority of studies enrolled patients with advanced disease, van den Borst et al. (2013) demonstrated that endurance is already impaired in mild-to-moderate COPD. As expected, patients with advanced disease suffer from greater impairment in endurance; for instance, Serres et al. (1998) reported a positive correlation between muscle endurance and FEV_1_. Nevertheless, other investigations failed to reproduce these results (e.g., Couillard et al., 2003; Allaire et al., 2004). Although muscle endurance and physical activity correlated in Serres et al. (1998) and Gouzi et al. (2011) found that *quadriceps* endurance was 40% lower in COPD compared with healthy controls despite similar levels of physical activity in daily life.

Supporting evidence for impaired muscle endurance can also be inferred by studies showing elevated muscle fatigability (Table 1). Using magnetic stimulation of the femoral nerve, Mador et al. (2000) showed that ∼60% of COPD patients developed a significant amount of contractile fatigue (i.e., a >15%-reduction in twitch force compared to baseline) following high-intensity cycling exercise to symptom limitation. The prevalence of contractile fatigue in COPD almost doubles with the use of more sensitive indexes, such as the potentiated twitch (81%) as opposed to the unpotentiated twitch (48%) (Mador et al., 2001). Although some amount of post-exercise contractile fatigue is expected in health (Polkey et al., 1996), the key point relates to the fact that a greater amount of contractile fatigue is seen in patients exposed to equivalent muscle “load” (i.e., relative work rate and exercise duration) and metabolic demand (Mador et al., 2003a). When exposed to a more relevant activity for daily life (walking), muscle fatigability is also higher in distal leg muscles (dorsi- and plantar flexors) (Gagnon et al., 2013). Table 1 depicts an overview of the results from the most prominent studies investigating muscle fatigability in patients with COPD (Mador et al., 2000, 2001, 2003a; Man et al., 2003; Saey et al., 2003, 2005; Butcher et al., 2009; Burtin et al., 2012; Gagnon et al., 2013).

**TABLE 1:** Outline of the main studies using magnetic stimulation of the femoral nerve to assess the presence (usually >15% reduction in the twitch (Tw) force) and severity of exercise-induced locomotor muscle fatigue in patients with chronic obstructive pulmonary disease (COPD).

**Author, year of publication**	**Study sample**	**Study design, intervention and muscle fatigue outcomes**	**Main results**	**Results interpretation**
**Contribution of muscle fatigue to exercise limitation**				
Mador et al., 2000	19 patients FEV_1_ = 42 ± 3% pred	Single-group study CWR cycling exercise test (60–70% WR_peak_) to Tlim Quadriceps unpotentiated Tw	↓ Quadriceps Tw post-exercise 10 min = 79.2 ± 5.4% 30 min = 75.7 ± 4.8% 60 min = 84.0 ± 5.0% of baseline value 11/19 patients were “fatiguers”	Locomotor muscle fatigue is present after CWR exercise to the limit of tolerance
Saey et al., 2003	18 patients FEV_1_ = 38 ± 14% pred	Single-group, randomized, crossover study CWR cycling exercise test (80% WR_peak_) to Tlim Randomly receiving either placebo or bronchodilators (500 μg ipratropium bromide) Quadriceps potentiated Tw	↑ Endurance time with bronchodilators only in the 9 “non-fatiguers” patients after placebo exercise Inverse correlation between ↑ Endurance time with bronchodilators and muscle fatigue after exercise with placebo	Locomotor muscle fatigue can contribute to exercise tolerance as bronchodilation fails to improve exercise tolerance in some patients
**Comparison of muscle fatigue between patients and controls**				
Mador et al., 2003a	9 patients FEV_1_ = 36 ± 5% pred 9 healthy controls	Controlled study Patients: CWR cycling exercise test (60% WR_peak_) to Tlim Controls: CWR cycling exercise test of similar duration and metabolic demand Quadriceps potentiated Tw	↓ Quadriceps Tw post-exercise in both groups at any time-point ↓ Quadriceps Tw post-exercise in patients *geq* controls at any time-point (10, 30, and 60 min)	Patients have greater locomotor muscle fatigability compared to healthy controls
Mador et al., 2003b	11 patients with mild- to-moderate COPD FEV_1_ = 50 ± 3% pred 8 patients with severe COPD FEV_1_ = 26 ± 2% pred 10 healthy controls	Controlled study contrasted by disease severity 3 sets of 10 MVC (5 s on/off) 3 min rest between sets quadriceps potentiated Tw	↓ Quadriceps Tw post-exercise in the 3 groups at any time-point (10, 30, and 60 min) ↓ Quadriceps Tw post-exercise in severe patients > mild-to-moderate patients and controls	Severe patients have greater muscle fatigability compared to controls mild-to-moderate patients have intermediate muscle fatigability compared to the other two groups
**Underlying mechanisms of muscle fatigue**				
Saey et al., 2005	32 patients stratified as 22 “fatiguers” FEV_1_ = 43 ± 14% pred 10 “non-fatiguers” FEV_1_ = 39 ± 15% pred	Single-group study CWR cycling exercise test (80% WR_peak_) to Tlim Muscle biopsies of the vastus lateralis muscle quadriceps potentiated Tw	↑ Lactate dehydrogenase activity, ↓ muscle capillarization and ↑ arterial lactate concentration after exercise in “fatiguers” vs. “non-fatiguers” Correlation between muscle fatigue and abovementioned parameters	A greater reliance on glycolytic metabolism during exercise is associated with muscle fatigability
Butcher et al., 2009	11 patients FEV_1_ = 52 ± 17% pred	Single-group, randomized, crossover study CWR cycling exercise test (80% WR_peak_) • to Tlim randomly breathing either room air or heliox (79% helium, 21% oxygen) for the first two test • to Tlim under room air but breathing heliox (isotime measurements) for the third test Quadriceps potentiated Tw	Under room air: ↓ Quadriceps Tw inversely correlated with end-exercise EELV Under heliox: ↑ exercise time inversely correlated with ↓ Quadriceps Tw under room air ↓ mechanical respiratory constraints at isotime room air	Patients with higher ventilatory limitations under room air showed lower muscle fatigue Exercise tolerance increased to a greater extent in these patients when breathing heliox due to delayed respiratory constraints, which eventually caused greater muscle fatigue at symptom limitation
**Muscle fatigue in response to walking-based exercise**				
Man et al., 2003	77 patients FEV_1_ = 41 ± 15% pred A subset of 12 patients FEV_1_ = 36 ± 11% pred performed muscle fatigue investigation	Single-group, randomized, crossover study Incremental + endurance walking (80% VO_2peak_) and cycling (80% WR_peak_) exercise tests to Tlim predominant limiting symptom determination quadriceps potentiated Tw after incremental walking and cycling exercise	Breathlessness alone=more common limiting symptom after incremental walking vs. cycling (81 vs. 34%) and endurance walking vs. cycling (75% vs. 29%) ↓ Quadriceps Tw post-exercise cycling > walking and only significant after cycling	Leg discomfort and quadriceps muscle fatigue are more frequent after cycling than walking
Gagnon et al., 2013	15 patients FEV_1_ = 54 ± 16% pred 15 healthy controls	Controlled study Endurance walking (12-min treadmill exercise with a fixed total expense of 40 Kcal) Dorsiflexors, plantar flexors and quadriceps potentiated Tw	Quadriceps Tw did not ↓ post-exercise in both groups ↓ Dorsi- and plantar flexors Tw post-exercise in patients ↑ healthy controls	Patients have greater distal leg muscles fatigability compared to healthy controls during walking
**Pulmonary rehabilitation and muscle fatigue**				
Mador et al., 2001	21 patients FEV_1_ = 45 ± 4% pred	Single-group study Pulmonary rehabilitation: endurance training (8 weeks, 3 sessions/week) CWR cycling exercise test (37 ± 4 W) to Tlim before PR (isotime measurements) quadriceps potentiated Tw	↓ Quadriceps Tw 10 min post exercise before PR: 74 ± 4%; after PR: 85 ± 4% of baseline value ↓ Quadriceps Tw post-exercise before PR ↑ after PR at any time-point (10, 30 and 60 min)	Pulmonary rehabilitation improves muscle fatigability in the quadriceps
Burtin et al., 2012	46 patients FEV_1_ = 42 ± 13% pred	Single-group study Pulmonary rehabilitation: Combined endurance and resistance training (3 months, 3 sessions/week) Determination of the presence of muscle fatigue after an exercise training session Quadriceps potentiated Tw	29/46 patients developed exercise training-induced muscle fatigue These “fatiguers” showed larger increase in 6-min walk distance and Chronic Respiratory Disease Questionnaire score after PR compared to “non-fatiguers” counterparts	Patients who developed muscle fatigue during exercise training showed greater training effects in terms of functional exercise capacity and health-related quality of life

*CWR, constant work rate; FEV_1_, forced expired volume in 1 s; MVC, maximal voluntary contraction; PR, pulmonary rehabilitation; Tlim, time to intolerance; VO_2peak_, peak oxygen uptake.*

### Summative Evidence

Impaired muscle protein synthesis/degradation ratio leading to variable degrees of muscle atrophy may underlie muscle weakness in COPD. However, the latter might be worse than expected by loss of muscle mass alone – at least in patients with relatively preserved lean body mass. Lower muscle endurance and exaggerated fatigability may stem from mitochondrial abnormalities, a low proportion of fatigue-resistant fibers and, in some patients, impaired capillarization. A note of caution should be made regarding the fact that the bulk of the evidence comes from *quadriceps*-based studies involving cycling, an exercise modality that taxes the appendicular muscles to a level which most patients are unlikely to face in daily life (Pepin et al., 2005; Marquis et al., 2009).

An extant and critical interrogation is whether impairments in locomotor muscle structure and function in COPD are entirely explained by muscle disuse due to physical inactivity or whether factors inherent to COPD can also be involved. As exposed in the preceding sections, recent investigations suggest the implication of specific factors to COPD (e.g., tobacco-smoke exposure) in muscle structural and functional abnormalities (Amann et al., 2010; Konokhova et al., 2016; Barreiro and Jaitovich, 2018; Gifford et al., 2018). As muscle disuse holds an indisputable role in muscle dysfunction in COPD (Jaitovich and Barreiro, 2018), rehabilitative exercise training is therefore the most rational mean to tackle these abnormalities.

## Can Locomotor Muscles Abnormalities Be Reversed by Exercise Training?

### Muscle Milieu

#### Oxidative Stress and Antioxidant Capacity

Exercise training has only limited beneficial effect on markers of oxidative and nitrosative stress in patients with COPD (De Brandt et al., 2016). In fact, several studies have shown an unchanged antioxidant capacity following aerobic (Barreiro et al., 2009) and high-intensity interval training [e.g., ∼ 90% peak work rate (WR_*peak*_) (Rabinovich et al., 2001)]; of note, antioxidant capacity was improved in healthy subjects after the same intervention (Rabinovich et al., 2001; Barreiro et al., 2009). In contrast, a recent investigation reported, for the very first time, an increase in muscle superoxide dismutase content after both endurance and resistance training, potentially leading to an enhanced clearance of ROS (Ryrso et al., 2018). Of note, cachectic patients with COPD may be particularly prone to deleterious exercise training-induced oxidative and nitrosative stresses: a reduction in antioxidant capacity (Rabinovich et al., 2006) and an increase in protein nitration (Vogiatzis et al., 2010) have been specifically reported following intervention in this subpopulation. Actually, these adverse processes likely hold a prominent role in skeletal muscle wasting in patients with COPD (Simoes and Vogiatzis, 2018). In a recent randomized controlled trial, antioxidant supplementation provided additional effects to rehabilitative exercise training alone on muscle structure and function (e.g., greater gains in type I muscle fiber proportion, antioxidant deficits and muscle strength) although muscle endurance improved similarly in both groups (Gouzi et al., 2019). This study is the first to suggest that efficient antioxidant supplementation results in further adaptations not explained by training alone in COPD.

#### Muscle Inflammation

Endurance training, either continuous constant-load [(Ryrso et al., 2018); 60% WR_peak_ (Vogiatzis et al., 2007)] or high-intensity interval training [100% WR_peak_ (Vogiatzis et al., 2007); ∼ 90% WR_peak_ (Rabinovich et al., 2003)], did not modify the mRNA or protein expression of different pro-inflammatory cytokines in COPD (Rabinovich et al., 2003; Vogiatzis et al., 2007; Ryrso et al., 2018). Although baseline values were ∼6 times greater in COPD, muscle TNF-α mRNA expression was not altered by exercise training in controls (Rabinovich et al., 2003). Comparing the effect on endurance and resistance training on muscle inflammation, Ryrso et al. (2018) found that both training modalities did not alter the content of pro-inflammatory cytokines and inflammatory cells. This suggests that exercise-based interventions, at least, does not worsen muscle inflammation (De Brandt et al., 2016) – if present (see section“Inflammation”). This assertion should be tempered with the findings of Menon et al. (2012b) who reported that 8 weeks of high-intensity resistance training resulted in a large reduction (↓100%) of exercise-induced neutrophils in the *quadriceps*. Muscle neutrophils were actually undetectable in the majority of patients, with no residual difference with controls as compared to pre-intervention.

### Muscle Micro-Structure

#### Mitochondria

Twelve weeks of endurance training (at WR eliciting 80% of peak oxygen uptake) successfully raised CS and hydroxyacyl-coenzyme A dehydrogenase (involved in fatty acid oxidation) activities in GOLD III patients, leading to less exercise-induced acidosis (Maltais et al., 1996). Similar results were achieved after shorter endurance training protocol [6 weeks starting at 70% WR_peak_ (Puente-Maestu et al., 2003)] or in response to combined endurance and resistance training (Gosker et al., 2006). Nevertheless, a study involving a similar training regimen failed to improve CS and lactate dehydrogenase activities in hypoxemic patients with COPD, suggesting that hypoxemia may hamper mitochondrial adaptation to training (Costes et al., 2015). Localized exercise training may also prove particularly useful: a 6-week knee extensor high-intensity interval training (90% WR_peak_) increased CS activity in the *quadriceps* in association with significant increases in peak O_2_ uptake and mitochondrial respiration (Bronstad et al., 2012). Finally, single-leg cycling may facilitate muscular adaptations to training: for instance, Abbiss et al. (2011) reported greater improvement in oxidative potential (e.g., cytochrome c oxidase concentration) of the skeletal muscle as compared to conventional cycling (both performed as intervals at self-paced maximal intensity). Short interventions (2-week duration) of single-leg cycling were sufficient to improve mitochondrial function [e.g., raising CS activity (Vincent et al., 2015; MacInnis et al., 2017)] in healthy participants, while intervals (65% WR_peak_) elicited larger improvements than constant-load modality [50% WR_peak_, (MacInnis et al., 2017)]. To the best of our knowledge, muscle adaptations to single-leg cycling have not been specifically investigated in COPD.

#### Muscle Protein Synthesis/Breakdown

Exercise training may modify the balance between myogenesis, protein synthesis and protein breakdown in favor of an exercise-induced anabolism in COPD (Simoes and Vogiatzis, 2018). In severe-to-very severe patients (GOLD stage III or IV), resistance training increased protein expression for anabolism, myogenesis and transcription factors – albeit at less extent compared to controls except for myogenesis (Constantin et al., 2013). In COPD patients with low plasmatic testosterone, testosterone plus resistance training was superior to resistance training alone in enhancing molecular adaptations signaling for anabolism e.g., increased mRNA for myosin heavy chain 2A and muscle IGF-I protein expression (Lewis et al., 2007). This was translated into a greater gain in muscle mass in the testosterone-supplemented group compared to resistance training alone. Improvements in muscle strength (+27% vs. +17%) and endurance/fatigability (+81% vs. +45%) also tended to exceed those observed in the resistance training alone (Casaburi et al., 2004). Endurance training (either constant-load or high-intensity interval training) also provided upregulation of pathways for muscle hypertrophy and regeneration [e.g., greater *quadriceps* IGF-I and MyoD protein expression (Vogiatzis et al., 2007)]. Myogenesis adaptations, however, were found to be abrogated in cachectic patients with COPD after endurance training [performed as intervals at 100% WR_peak_ (Vogiatzis et al., 2010)]; in fact, Atrogin-1 and MURF-1 (involved in muscle proteolysis) increased in the cachectic subgroup. In contrast, IGF-I and myostatin protein expression increased and decreased, respectively, in non-cachectic subjects (Vogiatzis et al., 2010). Combined endurance (performed at the ventilatory threshold or 60% WR_peak_) and resistance training had a non-significant increase in the activation of Akt/mTOR pathway in normoxemic, but not in hypoxemic, patients (Costes et al., 2015). Actually, greater beneficial changes in muscle molecular responses to rehabilitative exercise training were recently associated with larger gains in exercise capacity in COPD (Kneppers et al., 2019). Overall, the fact that cachectic and hypoxemic patients with COPD showed different response to training than their respective non-cachectic and normoxic counterparts, specific management in the frailer patients might be necessary to trigger induce positive muscle adaptations [e.g., nutritional ergogenic aids (Fuld et al., 2005; Villaca et al., 2006) or blockade of negative muscle regulators (Polkey et al., 2019) in selected patients]. In addition, the fact that differences in atrophy/hypertrophy signaling pathways in COPD and controls are observed after accounting for medication, aging and physical inactivity (Doucet et al., 2007) and that rehabilitative exercise training fails to restore, partially (Constantin et al., 2013) or completely (Vogiatzis et al., 2010; Costes et al., 2015), the balance between muscle protein synthesis and breakdown in these patients suggest that COPD may hold a specific role in the observed alterations, in addition to physical inactivity *per se*.

### Muscle Macro-Structure

Using biopsies of the *vastus lateralis* muscle, an increase in muscle fiber size [+12–21%, (De Brandt et al., 2016)] has been reported following combined aerobic and resistance training (Costes et al., 2015), high-intensity interval training (e.g., Vogiatzis et al., 2010) and neuromuscular electrical stimulation (Dal Corso et al., 2007). Conversely, muscle fiber size was unchanged in hypoxemic patients with COPD after combined endurance/resistance training (Costes et al., 2015) or even reduced following endurance training [performed at the ventilatory threshold (Gouzi et al., 2013b)]. This impairment was not observed in controls after the same intervention (Gouzi et al., 2013b). An increase in type II muscle fiber size of similar magnitude between COPD and controls was observed after 8 weeks of resistance training alone (Menon et al., 2012b).

A decrease in *vastus lateralis* type IIx muscle fiber proportion has been reported in COPD after a 10-week cycling endurance [either constant-load (60–80% WR_peak_) or intervals (100–140% WR_peak_)] training (Vogiatzis et al., 2005). Conversely, fiber distribution remained unchanged in patients after two different 12-week cycling endurance training programs [80% WR_peak_ (Whittom et al., 1998); 50–80% WR_peak_ (Guzun et al., 2012)] but also in healthy controls (Guzun et al., 2012). Ten weeks of bicycle-based high-intensity interval training (80–100% WR_peak_) increased *vastus lateralis* type I muscle fiber proportion in GOLD II and IV COPD patients (Vogiatzis et al., 2011). Interval training, in particular, has been shown to be effective in reducing type IIx fiber proportion in the above-mentioned muscle across all COPD stages (Vogiatzis et al., 2005, 2007, 2011) but the increase in type I fiber proportion is not a universal finding (Vogiatzis et al., 2005, 2007). Interestingly, *vastus lateralis* muscle fiber type remodeling was also present in a group of cachectic patients with COPD (Vogiatzis et al., 2010). Conversely, no significant change in fiber type proportion in the *quadriceps* was found after resistance training (Lewis et al., 2007; Iepsen et al., 2016). Similarly, combined endurance and resistance training failed to modify fiber type distribution in COPD (Gosker et al., 2006; Gouzi et al., 2013b; Costes et al., 2015) but not in controls (Gouzi et al., 2013b).

Improvement in *vastus lateralis* capillary-to-fiber ratio has been demonstrated after constant-load cycling (Vogiatzis et al., 2005), high-intensity interval training (Vogiatzis et al., 2005, 2010) and combined endurance and resistance training (Costes et al., 2015; Gouzi et al., 2016) in COPD. When compared to healthy controls, the extent of improvement was ∼ halved in patients with COPD (Gouzi et al., 2016). However, the improvement in *vastus lateralis* capillarization is not consistent; for instance, endurance (Whittom et al., 1998; Iepsen et al., 2016) or resistance (Iepsen et al., 2016) training failed to alter this variable in some studies. It should be noted that improvement in muscle capillarization can be hindered in specific subgroups of patients, such as those presenting with significant hypoxemia (Costes et al., 2015).

### Muscle Mass

Muscle mass increased in the lower limbs (+8.5%) following an 8-week walking-based endurance training program (Farias et al., 2014) but this was not found after 12 weeks of constant-load (80% WR_peak_) endurance cycling (Bernard et al., 1999). Beyond the different mode of exercise, participants performed 5 vs. 3 weekly sessions, respectively, while each session was twice longer in the former study (i.e., up to 60 min vs. 30 min). This may have led to diverging exercise-induced benefits in terms of muscle mass between the two studies. In contrast, resistance training consistently increased muscle mass [∼5–20%, (Menon et al., 2012a, b)], being more effective than endurance training to counteract muscle atrophy. These gains were of similar magnitude than those observed in healthy controls (Menon et al., 2012a, b). Skeletal muscle mass was also found to improve [+ 8% in thigh cross sectional area as compared to baseline assessment (Bernard et al., 1999)] when endurance and resistance modalities are combined. In severe COPD patients presenting with incapacitating breathlessness on minimal exertion, neuromuscular electrical stimulation (NMES) may be a valuable substitute to increase muscle mass (Maddocks et al., 2016). However, early NMES (i.e., before muscle mass wasting ensues) might lead to better results (Napolis et al., 2011).

### Muscle Strength

Following endurance training alone, isometric *quadriceps* strength increased by 10 to 21% among studies (De Brandt et al., 2018). However, when data from healthy controls are available, endurance training failed to improve isometric *quadriceps* strength in both groups (Guzun et al., 2012). Isotonic *quadriceps* strength also improved after endurance training by ∼ 8 (Bernard et al., 1999) to 20% [(Ortega et al., 2002): intensity 70% WR_peak_] in COPD with no available comparison with controls. However, such beneficial effects are not uniformly reported [e.g., Mador et al., 2004: initial intensity 50% WR_peak_]. Another investigation (80% WR_peak_) reported a 14%-improvement in isokinetic strength in COPD while no change was observed in controls (Radom-Aizik et al., 2007). Non-volitional un-potentiated and potentiated twitch force also increased after this training modality in COPD (Mador et al., 2001). After demonstrating its feasibility in COPD (Rocha Vieira et al., 2011), MacMillan et al. (2017) recently reported an improvement in *quadriceps* maximal isometric strength (and relative thigh muscle mass) after a 10-week eccentric cycle training program while no change was observed in the conventional exercise group. In fact, exertional symptoms in the eccentric exercise group were lower despite participants exercised against a 3-time greater resistance as compared to conventional exercise modality. Eccentric cycling may, therefore, be a valuable alternative to the conventional concentric modality to facilitate exercise-induced muscle adaptations in COPD.

As recently reviewed by De Brandt et al. (2018), resistance training is a particularly effective modality to improve the different muscle strength outcomes (isometric, isokinetic and isotonic strength) in patients with COPD. Isotonic strength of the *quadriceps*, for instance, increased up to 53% after 12 weeks of this training modality (Ortega et al., 2002). When investigations included a group of healthy controls undergoing resistance training, the gains in muscle strength were, at least, of similar magnitude in patients with COPD (Menon et al., 2012a, b). Combining endurance and resistance modalities led to a gain in maximal *quadriceps* strength in COPD and healthy controls, with no significant difference between groups in terms of magnitude (Gouzi et al., 2013b). In patients presenting with advanced respiratory mechanical and pulmonary gas exchange impairments, symptom-targeted exercise intensity and/or localized passive training [e.g., NMES (Neder et al., 2002; Vivodtzev et al., 2012)] might be the only feasible alternative to obtain some (minor) improvement in peripheral muscle strength.

### Muscle Endurance and Fatigability

Data regarding isolated muscle endurance after training in patients with COPD are scarce; however, some few studies reported an increase of 50–60% after 4–8 weeks of aerobic exercise [∼ 40–65% WR_peak_ (Vivodtzev et al., 2010); 50–80% WR_peak_ (Covey et al., 2014)]. Muscle endurance also increased after endurance-oriented resistance training using low-load elastic bands [+10%, (Nyberg et al., 2015)] or simply body mass (Clark et al., 1996). Similarly, combined endurance and resistance training provided significant improvement in muscle endurance; nevertheless, its effect varied substantially among studies [from 20% to almost 100%, (Franssen et al., 2005; Gouzi et al., 2013b; Covey et al., 2014); initial intensity 50–60% WR_peak_ in Franssen et al. (2005)]. The magnitude of improvement in muscle endurance was, however, lower in patients with COPD as compared to healthy controls (Gouzi et al., 2013b). Improvement in markers of oxidative metabolism was actually blunted or even absent in COPD which likely explains the lower functional gain observed in patients (Gouzi et al., 2013b). Of note, although both endurance training alone and combined endurance/resistance training substantially increased muscle endurance in Covey et al. (2014), the magnitude of improvement was twice larger in the combined modalities group. Using a non-volitional technique, Mador et al. (2001) found a blunted decrease in potentiated twitch force post- compared to pre-rehabilitation for the same intensity and duration of exercise, indicting less *quadriceps* fatigability (Table 1). Exercise training-induced improvement in muscle O_2_ delivery and utilization may have contributed to this beneficial changes (Barberan-Garcia et al., 2019). Of note, downhill walking may also prove useful: it has been shown to induce *quadriceps* muscle fatigue (Camillo et al., 2015) which is associated with larger improvement in exercise capacity and quality of life after rehabilitative exercise training (Burtin et al., 2012).

### Summative Evidence

There is little evidence that rehabilitative exercise training significantly improves the derangements in muscle milieu in patients with COPD. Nevertheless, beneficial changes in muscle structure and function can be elicited particularly an increase in mitochondrial activity/number and increased activity of anabolic pathways. Hypoxemia, however, dampens improvement in oxidative metabolism and muscle endurance gain in COPD. Exercise-induced changes in atrophy/hypertrophy signaling pathways were also abrogated in the presence of hypoxemia or cachexia. In these specific patients, the effect of negative muscle regulators’ blockade as an adjunct to rehabilitative exercise training might be investigated in upcoming trials in order to facilitate positive muscle adaptations. The safety and tolerability of this intervention alone has been recently established in patients with COPD.

As expected, resistance is more effective than endurance training to improve muscle mass and strength. To the extent that the literature permits, these gains appear of similar magnitude as compared to healthy controls. Endurance training (either alone or in combination with resistance training) or high-intensity interval training may improve muscle capillarization in selected patients. The latter training modality, in particular, seems to constitute the most efficient intervention to reverse type I-to-II muscle fiber shift, likely due to less “central” (i.e., mechanical-ventilatory) constraints to exercise tolerance. Surprisingly, only a limited number of investigations included healthy controls undergoing rehabilitative exercise training. Consequently, it remains unknown what is the comparative extent of improvement for a substantial number of muscle-centered outcomes in COPD. Future studies should pay attention to enroll well-matched healthy controls to address this concern.

## Conclusion

The current concise review found robust evidence that beneficial changes in muscle characteristics and function may be obtained with rehabilitative exercise training in most (but not all) patients with COPD without triggering additional deleterious consequences such as local oxidative stress and/or inflammation. In patients who can tolerate sufficiently high training intensities, a combination of dynamic exercise (notably interval-based) and resistance training are particularly effective. Hypoxemia and cachexia, however, are disease traits that predict lower responses to training. In these patients and other subpopulations with more advanced disease, alternative exercise training modalities might prove useful, including NMES, single-leg or eccentric exercise, water-based training and others. In any case, the ultimate challenge of pulmonary rehabilitation (whose rehabilitative exercise training is a single but essential component) is to provide effective strategies to ensure that eventual improvements in functional capacity are translated into enhanced levels of daily physical activity. Therefore, future research should focus on educational interventions promoting long-term behavioral and lifestyle changes, as improvements obtained during rehabilitative exercise training are poorly retained over time in patients with COPD.

## Author Contributions

MM and A-CB reviewed the relevant literature on the topic and drafted the manuscript. SV and JN provided the critical feedback to shape the final version of the manuscript. All authors contributed significantly to the present work.

## Conflict of Interest

The authors declare that the research was conducted in the absence of any commercial or financial relationships that could be construed as a potential conflict of interest.
